# Forward Genetic Screening for the Improved Production of Fermentable Sugars from Plant Biomass

**DOI:** 10.1371/journal.pone.0055616

**Published:** 2013-01-31

**Authors:** George Stamatiou, Danielle P. Vidaurre, Isaac Shim, Xurong Tang, Wolfgang Moeder, Dario Bonetta, Peter McCourt

**Affiliations:** 1 Department of Cell & Systems Biology, University of Toronto, Toronto, Ontario, Canada; 2 Faculty of Science, University of Ontario Institute of Technology, Oshawa, Ontario, Canada; University of Massachusetts Amherst, United States of America

## Abstract

With their unique metabolism and the potential to produce large amounts of biomass, plants are an excellent bio-energy feedstock for a variety of industrial purposes. Here we developed a high-throughput strategy, using the model plant *Arabidopsis thaliana*, to identify mutants with improved sugar release from plant biomass. Molecular analysis indicates a variety of processes including starch degradation, cell wall composition and polar transport of the plant hormone auxin can contribute to this improved saccharification. To demonstrate translatability, polar auxin transport in maize was either genetically or chemical inhibited and this also resulted in increased sugar release from plant tissues. Our forward genetic approach using Arabidopsis not only uncovers new functions that contribute to cell wall integrity but also demonstrates that information gleaned from this genetic model can be directly translated to monocotyledonous crops such as maize to improve sugar extractability from biomass.

## Introduction

**Figure 1:**
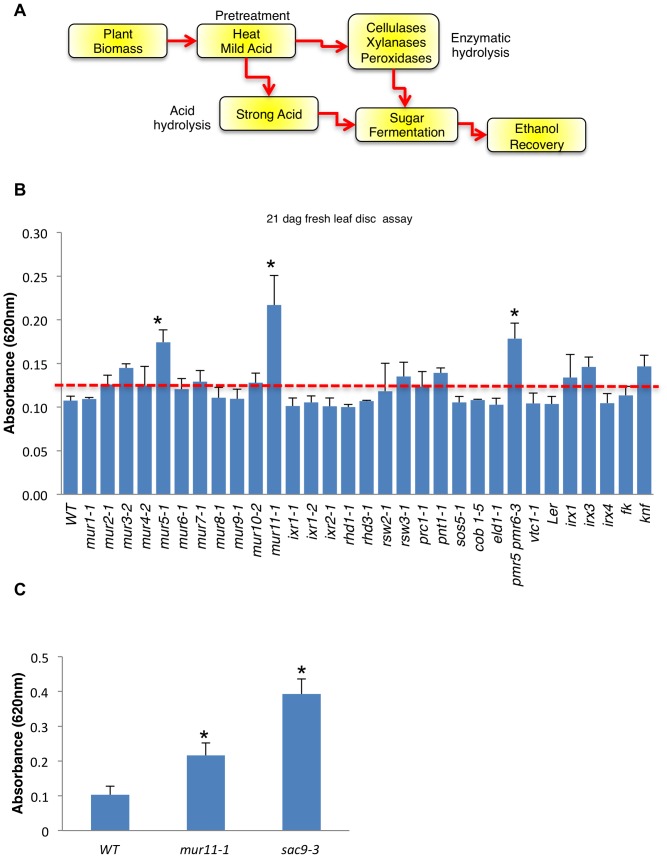
Screening for *wall hydrolysis sensitive (rah)* mutants. (A) Schematic of the production of ethanol from cellulosic biomass. For biomass pretreatment, dilute sulphuric acid is used to solubilize the hemicellulosic fraction and to disrupt the crystalline structure of cellulose so that hydrolyzing enzymes can easily access and convert cellulose to fermentable sugars. (B) Leaf discs of known cell wall mutants were subjected to acid hydrolysis using 1 M H_2_SO_4_ at 21 dag. Of the 30 cell wall mutants tested *mur11-1* showed a significant difference in saccharification relative to wild type. Graph shows absorbance at 620 nm for ¼ of a leaf disc hydrolysate; values are averages ± s.d. (*n* = 4). We repeated all experiments at least three times with similar results. Dotted red line denotes 2 standard deviations above wild type levels. (C) Fresh leaf disc tissue of *mur11-1* and a T-DNA allele of *MUR11*, *sac9-3* (SALK_058870), were assayed for increased saccharification using 1 M H_2_SO_4_ at 21 dag. Graph shows absorbance at 620 nm for ¼ of a leaf disc hydrolysate; values are averages ± s.d. (*n* = 8–10). *, P<0.01 using Student's *t*-test.

Plant biomass has gained considerable interest as a stable, environmentally benign source of energy [1]–[3]. However, much of the plant's biomass is encapsulated in the cell wall in the form of cellulose, and branched polysaccharides, collectively known as hemicellulose, and lignin. Moreover, the biochemical conversion of cell walls to a useful carbon source is a costly and energy inefficient process. This recalcitrance has led to the development of a variety of technologies that usually involve the deconstruction of plant cell walls through either acid, thermochemical and/or enzymatic hydrolysis (Fig. 1A). For example, hemicellulose can be hydrolyzed by dilute acid treatments but these conditions are not severe enough for cellulose hydrolysis [4], [5]. Increasing acid concentrations or carrying out acid treatments at high temperature and pressure improves sugar yields from cellulose but both processes are corrosive and increase costs. Unfortunately, enzymatic approaches of digesting cell wall material are not a mature technology. Moreover, the protective nature of the cell wall to cellulases means digestion is slow and inefficient. As a consequence, acid hydrolysis pretreatments are again often used to depolymerize and solubilize hemicelluloses. The lack of energy efficient and environmentally friendly conversion of cell wall polymers into fermentable sugars or saccharification has spurred interest in using genetic and genomic approaches that modify the cell wall for industrial processing [2], [3], [6]. Often these approaches have involved manipulating known cell wall synthesis or degradation enzymes. Although these rational approaches are promising, they depend on a prior molecular knowledge of the genes of interest usually followed by reverse genetics to test functionality. By contrast, forward genetic screens, which inherently have no mechanistic bias, have the potential to uncover novel processes that could improve saccharification. The limitation of forward screens, however, is designing specific high throughput assays, followed by efficient molecular identification of the genes involved. In this latter case, however, the recent development of next generation sequencing technologies to identify mutant alleles has greatly reduced this bottleneck [7], [8].

With these considerations, we use the model plant genetic system *Arabidopsis* to develop forward genetic screens for mutants with improved saccharification from plant tissues. Our screens were based on finding mutants that showed improved sugar release compared to wild type plants under mild acid hydrolysis conditions. Interestingly, the mutants that gave the best saccharification phenotypes mostly encoded genes that would not be thought to have any obvious role in cell wall biology. For example, we found that perturbing specific steps in starch metabolism and the transport of the plant hormone auxin improved sugar release from vegetative tissues. In this latter case, this technology could be translated to the biomass crop, *Zea mays*. These results indicate that many unknown processes influence cell wall integrity which in principle expand the potential genotypes that may contribute to improving industrial processing of plant biomass.

## Materials and Methods

### Plant materials and growth conditions

*Arabidopsis thaliana* ecotype Columbia M2 seeds mutagenized by ethyl methane sulfonate (EMS) were purchased from Lehle Seeds (Round Rock, TX). EMS mutant alleles and T-DNA insertions were provided by the *Arabidopsis* Biological Resource Centre (Ohio State University, Columbus, USA). Seeds were surface sterilized in 50% bleach, 0.01% Tween-20 for 5 min, rinsed 5 times with sterile water and stored in the dark at 4°C for 4 days to synchronize germination. Seeds were plated on 0.5× strength Murashige and Skoog (MS) agar plates and sealed with surgical tape under continuous light at room temperature. Plants were grown to senescence (∼8 weeks) by sowing them onto a mixture of sphagnum peat moss, perlite and vermiculite (Promix) and incubated in a growth chamber under continuous illumination (200 µE/m^2^/s) at 21°C. The maize mutants, *bif2-N2354* (stock #108A) and *bal* (stock #318B) in the W23/M14 genetic background, were obtained from the Maize Genetics Cooperation Stock Center.

### Acid sensitive mutant screen

**Figure 2:**
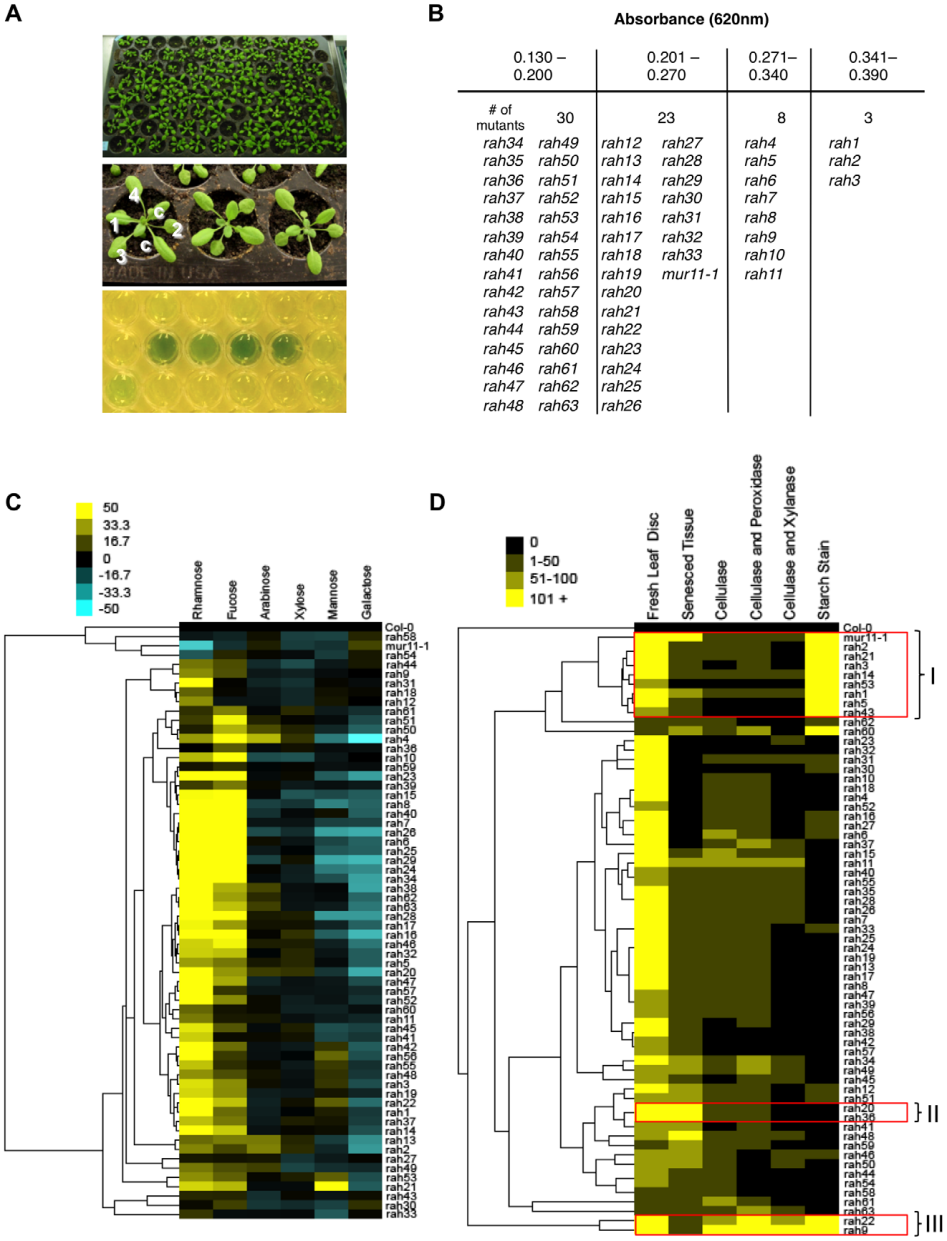
Characterization of *rah* mutants. (A) Three-week old *Arabidopsis* plants grown in 96-well flats at 22°C under a 16 h/8 h light/dark cycle (*top panel*). Leaf 3 or 4 was excised from 21 day-old plants using a hole punch and subjected to acid hydrolysis using 1 M H_2_SO_4_ (*middle panel*). c, cotyledon; leaf numbers indicated. Results of colorimetric anthrone assay illustrating that *rah* mutants release more sugars and turn a blue/green colour. Yellow indicates baseline levels of sugar release (*bottom panel*). (B) From the screen, 63 *rah* mutants and *mur11-1* were isolated and organized into 4 categories of low to high yielding sugar mutants. (C) Hierarchical cluster analysis of monosaccharide composition analysis by gas chromatography of *rah* mutants in 21 day-old seedlings. Values shown as a percentage relative to wild type (see Methods). Yellow indicates higher levels than wild type; blue, lower levels. (D) Clustered heatmap of sugar content as measured by the anthrone method from 63 *rah* mutants subjected to acid hydrolysis of fresh leaf tissue using 1 M H_2_SO_4_, acid hydrolysis of senesced stem tissue using 0.2 M H_2_SO_4_, or glucose content (using hexokinase method) of cellulase, cellulase+xylanase and cellulase+peroxidase digested senesced tissue and starch staining of 14 day-old seedlings. Values shown as a percentage relative to wild type (see Methods). Yellow indicates high levels of sugar; black, low levels of sugar. Mutants with similar characteristics have been classified into 3 subcategories (I, II and III) and outlined in red.

The M2 generation of EMS-mutagenized *Arabidopsis* (Col-0) seeds were chilled for 4 days and sown onto 0.5× MS plates placed vertically under continuous light conditions at room temperature. After 7 days, the seedlings were transferred to soil in 96-well flats. Leaf 3 or 4 was excised from 21 day-old plants using a hole punch and placed abaxial side up in a 96-well plate corresponding to the same coordinates as the flat. The hole punch produced a disc of uniform in size and weight; we purposely avoided the midvein to avoid thickness variability. Samples were submerged in 200 µl of 1 M H_2_SO_4_ and incubated at room temperature for 1 hour. A 50 µl aliquot was removed and incubated with 100 µl of 0.2% anthrone (Sigma-Aldrich, A91205) in concentrated (18 M) H_2_SO_4_. The samples were incubated at 100°C for 5 minutes, cooled and the absorbance was read at 620 nm [9]. Approximately 22,000 seedlings from 32 pools were screened from which 63 *responsive to acid hydrolysis* (*rah*) mutants were identified as having an absorbance reading greater than 2 standard deviations from wild type (see Fig. 2A and 2B; Fig. S1). *rah* mutants were retested in the M3 generation.

### Genetic and Physical Mapping of mutants

Genetic mapping was accomplished using an F2 population derived from a cross between the *rah* mutants (Columbia genotype, Col-0) and Landsberg *erecta* (L*er*). F2 seedlings were scored for acid hydrolysis sensitivity as determined by anthrone dependent colour development. Genomic DNA was isolated from individual F2 plants from a mapping population showing the mutant phenotype and assigned to a chromosome using published simple sequence length polymorphism (SSLP) markers (Fig. S3). New molecular markers were developed using the Monsanto Col-0 and L*er* polymorphism database. The cloned *RAH* genes were amplified by PCR using X-Taq DNA polymerase with proofreading activity (Takara). Sequencing reactions were performed by The Centre for the Analysis of Genome Evolution and Function (CAGEF) at the University of Toronto. F2 mutants from two independent crosses were used for sequencing and verifying lesions.

### Enzymatic Digestion

The senesced above-ground tissue (>95% is stem tissue) of *Arabidopsis* was prepared by initially drying it at 65°C for 2 days. The tissue was then ground using a Thomas Scientific Mini-Mill (Model 3383-L10) and passed through a 60 mesh screen. Approximately 100 mg of this ground tissue was then macerated in 10 mL of water at 20°C in glass test-tubes for three hours. The water was aspirated away and replaced with fresh 10 mL of water after which the tubes were placed in a water bath set at 80°C for one hour. The water was then exchanged with 10 mL of 70% ethanol and placed back in 80°C for an additional hour. The tissue was then rinsed twice with 5 mL of acetone, and the ground and washed tissue was oven dried at 65°C for 2 days. Cellulase (Celluclast, Sigma-Aldrich, C2730) and Cellobiase (Novozyme 188, Sigma-Aldrich, C6105) had activities empirically determined to be 111 filter paper units (FPU)/mL and 500 U/mL respectively. Cellulase activities were determined using the Filter Paper Assay [10], where the glucan products were quantified using 0.2% anthrone according to a glucose standard. The cellobiase activity was determined by assaying the *p*-nitrophenol formation from p-nitrophenyl-*1*,*4*-*β*-glucoside (Sigma-Aldrich, N7006) by measuring the absorbance at 400 nm. Triplicates of 15 FPU/g tissue of cellulase and 80 U/g of cellobiase were used on 5 mg of tissue in 200 µL of 50 mM sodium citrate, pH 4.8 in 1.5 mL microcentrifuge tubes. The samples were incubated in a water bath set at 50°C for 24 hours then heat inactivated at 100°C for 5 min. Once cooled on ice, the samples were centrifuged at 12 000 *g* for 5 min and the supernatant was retained. The free glucose in the supernatant was measured using a Glucose Hexokinase Assay Kit. (Sigma-Aldrich, GAHK20). Digestion of the same plant material with xylanase (Sigma-Aldrich X2753) was by incubating 5 mg of washed milled tissue in 50 mM sodium citrate buffer (pH 5.0) containing 0.57 U of xylanase, 0.077 U of cellulase and 0.4 U Novozyme 188 for 24 hours at 50°C. The reactions were terminated by heating at 100°C for 5 min. Samples were then centrifuged and the supernatant was assayed for the presence of glucose using the Glucose Hexokinase Assay Kit. In cases where lignin peroxidase (Sigma-Aldrich, 42603) was used, it was done prior to using the above enzyme mixtures by incubating the tissue in 50 mM sodium citrate buffer (pH 3) containing 0.00436 U peroxidase, 0.3% hydrogen peroxidase for 2 hours at 30°C.

### Gas-liquid chromatography

Hydrolysis of leaf material and quantification of monosaccharides by gas-liquid chromatography of alditol acetates was carried out as previously described [11]. At least 5–20 mg of fresh tissue from 5, 21-day old plant lines were pooled and extracted three times with chloroform∶methanol (1∶1) for 30 min each. Three technical replicates were performed for each *rah* mutant. The tissue was washed with 70% ethanol at 70°C for 1 hour, rinsed with acetone and left to air-dry overnight and hydrolyzed in 1 M H_2_SO_4_ at room temperature or at 120°C for 1 hour. The released monosaccharides were converted into alditol acetates and quantified by gas chromatography. Relative sugar composition values were calculated as mol percentages.

### Clustering and Heatmap analysis

The monosaccharide composition of 62 *rah* mutants (*rah35* not determined) and *mur11-1* was determined by liquid gas chromatography and calculated as a percent difference relative to wild type (Fig. 2C). Cluster 3.0 using the C Clustering Library version 1.49 was used to cluster the values by Average Linkage and centered correlation. Java TreeView 1.1.5r2 was then used to display the data and color-coded yellow (more than wild type) or blue (less than wild type). Sugar values quantified from the acid hydrolysis and enzymatic assays performed on the 63 *rah* mutants (Fig. 2D), excluding the starch staining, were calculated as a percent difference relative to wild type. Mutants with values equal to wild type were color coded black and mutants with sugar values greater than wild type were color coded yellow. For starch staining, 14 day-old seedlings were stained with iodine-potassium iodide (IKI) [12] and were visually analyzed for the presence of starch in their cotyledons and determined qualitatively. Qualitative staining for lignin was done by first growing seedlings for 10 days and decolorizing in 80% (v/v) ethanol. Cleared seedlings were stained and mounted with 1%(w/v) phloroglucinol (Sigma-Aldrich, P3502) in 6 M HCl and viewed under dark-field illumination.

### Amylase Digestion

Five milligrams of milled and washed senesced, stem tissue was weighed out in triplicate in 1.5 mL microcentrifuge tubes. The tissue was macerated in 940 µL of 0.1 M sodium acetate, pH 5 for several hours. The starch was then gelatinized by incubating the tubes at 80°C for 30 min. The tubes were cooled on ice, and then 30 µL of a tenfold dilution of α-amylase (Sigma-Aldrich A7595, undiluted activity: 250 U/mL) was added. In addition, 15 µL of pullulanase M1(Megazyme 699 U/mL) and 15 µL of pullulanase M2 (Megazyme 250 U/mL) were added to bring the total liquid volume to 1 mL. The samples were vortexed then placed in an incubator set at 37°C for 16 hours. The tubes were placed at 100°C for 5 min, cooled to room temperature then centrifuged at 12,000 *g* for 10 min. The reducing sugar equivalents present in the supernatant were quantified using 0.2% anthrone.

### *N*-1-naphthylphthalamic acid (NPA) treatment of monocot plants

Polar auxin transport inhibition was carried out as described [13]. The two maize cultivars, Syngenta hybrid N39-Q1 and Tuxedo Sweet Corn, were grown in a greenhouse at 24°C with a 12 hour day/night cycle. The plants were grown four weeks before NPA treatment followed by a two week watering regime using 120 µM NPA (ChemService, West Chester, Pennsylvania, USA) or DMSO alone (solvent) applied every two days in a volume of 150 mL for each pot (3.5″×3.5″). Plants were fertilized once a week with 150 mL of 20-20-20 fertilizer (5 g per gallon) with or without NPA. After 2 weeks of treatment, above ground tissue (mostly leaves and stems) was collected and destained in chloroform∶methanol (1∶1 v/v). The tissue was thoroughly dried in an oven and milled to a 60 mesh size. Acid hydrolysis was performed as described above.

## Results

### Screening for *responsive to acid hydrolysis* mutants

To work out a medium to high throughput screen for mutants that show altered sugar release required developing a simple reproducible assay that allows visualization of saccharification from plant tissue. Once putative mutants were identified they could be further tested using more quantitative methods such as gas chromatography to identify lines that had specific defects in cell wall composition. We settled on the anthrone assay as this reagent turns visually blue or green in the presence of sugars [9], [14]. Although absorption spectra vary depending on the sugar reacted with the anthrone reagent (see Fig. S2), this assay provides a convenient and simple first-pass measure of overall sugar content. We found that an Arabidopsis leaf punch incubated in dilute acid (1 M H_2_SO_4_) at room temperature for one hour gave an average reading of 0.12 absorbance units (±0.002, n = 100) at 620 nm (Fig. S1A). These samples developed a pale blue-green color (Figure 2A). With this baseline, we next applied our assay against a collection of 30 known cell wall mutants as indexed by the Plant Cell Wall Biosynthesis Research Network (WallBioNet) (Fig. 1B, Table S1). Although some in this collection are not annotated as having defects in leaf cell wall composition we thought the collection was a useful starting point to the development of our screen. Interestingly, of the 30 mutants tested, *mur5-1*, *mur11-1* and *pmr5*, *pmr6-3* showed the highest saccharification. We chose to further investigate *mur11-1* since it had the highest sugar release at approximately 2 fold greater relative to wild type. Map-based cloning of the *mur11-1* allele identified a transition mutation (G→A) in a conserved domain of the previously characterized gene, *SUPPRESSOR OF ACTIN9* (*SAC9*), which encodes a phosphoinositide phosphatase (Table S2) [7], [15]. This result was verified by demonstrating that another *mur11* allele, *sac9-3*, also showed improved sugar release by acid hydrolysis (Fig. 1C). With the finding that mutations in *SAC9* gave increased sugar release we decided to assay loss-of-function alleles of the complete *SAC* family of genes in *Arabidopsis* (*sac1-sac9*) [16]. We found, however, that no other *SAC* gene contributed to sugar release, which is perhaps not surprising since *SAC9* is only distantly related to the other *SAC* members of this family (Fig. S4)

The success of finding mutations that increase sugar release under our assay conditions in this indexed cell wall collection encouraged us to expanded the mutational space by applying our screen to a population of EMS-mutagenized *Arabidopsis* seedlings (Fig. 2A). At this stage, we limited our screen to rosette plants that showed no obvious growth or developmental defects since such defects at these early developmental stages may compromise the application value of the genes identified. From approximately 23,000 M2 plants representing 32 M1 parental groups we identified 63 mutants that showed increased saccharification (Fig. 2B). Designated *responsive to acid hydrolysis* (*rah*), we sub-categorized mutant lines into four groups based on the amount of sugar they released per fresh leaf disc (Fig. 2B).

The crude nature of the anthrone assay means the source of the increased saccharification is not clear. For example, it is possible that the mutation increases the amount of soluble sugar such as glucose through an alteration in photosynthesis. To determine if any of these mutants showed defects in cell wall sugars we performed gas chromatographic analysis of alditol acetates to identify changes in neutral sugar composition of the primary cell wall (Fig. 2C, Table S3). Interestingly many of the *rah* lines showed increases in rhamnose and fucose compared to wild type samples, which indicated that many of the mutations did perturb cell wall composition. Second, since our primary screen was performed on leaf tissue we wanted to determined if these defects in sugar release were also seen in other tissues and at later developmental times. This point is particularly important since most plant biomass for industrial purposes will come from stems of dried senesced crops. We therefore performed enzymatic hydrolysis of senesced, stem tissue using cellulase and cellobiase to assay cellulose hydrolysis; xylanase for hemicellulose break down; and a cocktail of cellulase, cellobiase, xylanase and peroxidase which, in addition to cellulose and hemicellulose, degrades lignin (Fig. 2D, Table S4). We also assayed the presence of starch in our samples, using IKI solution [12], as this source of carbon could potentially contribute to an increased sugar release phenotype in our assays. Finally, we also assayed senesced stem tissue hydrolyzed with 0.2 M sulphuric acid; an acid concentration that is more similar to industrial standards.

Hierarchical clustering of the various assays identified two main categories of mutants that we chose to focus on. One category consisted of two mutant lines (*rah20*, *rah36*) that showed increased sugar release with both fresh and senesced tissue acid hydrolysis. A second category consisted of nine lines (*mur11-1*, *rah1*, *rah2*, *rah3*, *rah5*, *rah14*, *rah21*, *rah43*, *rah53*) which hyperaccumulated starch (boxed areas in Fig. 2D). Another group in this category included two lines (*rah9* and *rah22*) which showed excess sugar release in all enzymatic assays and hyperaccumulated starch. Among the remaining mutant lines, nine showed ectopic lignin staining (see Table S4) while others did not show acid dependent saccharification in senesced tissues or high starch accumulation but did show increased sugar release by enzyme hydrolysis (Fig. 2D; Table S4).

### Specific genes involved in starch metabolism improve saccharification

**Figure 3:**
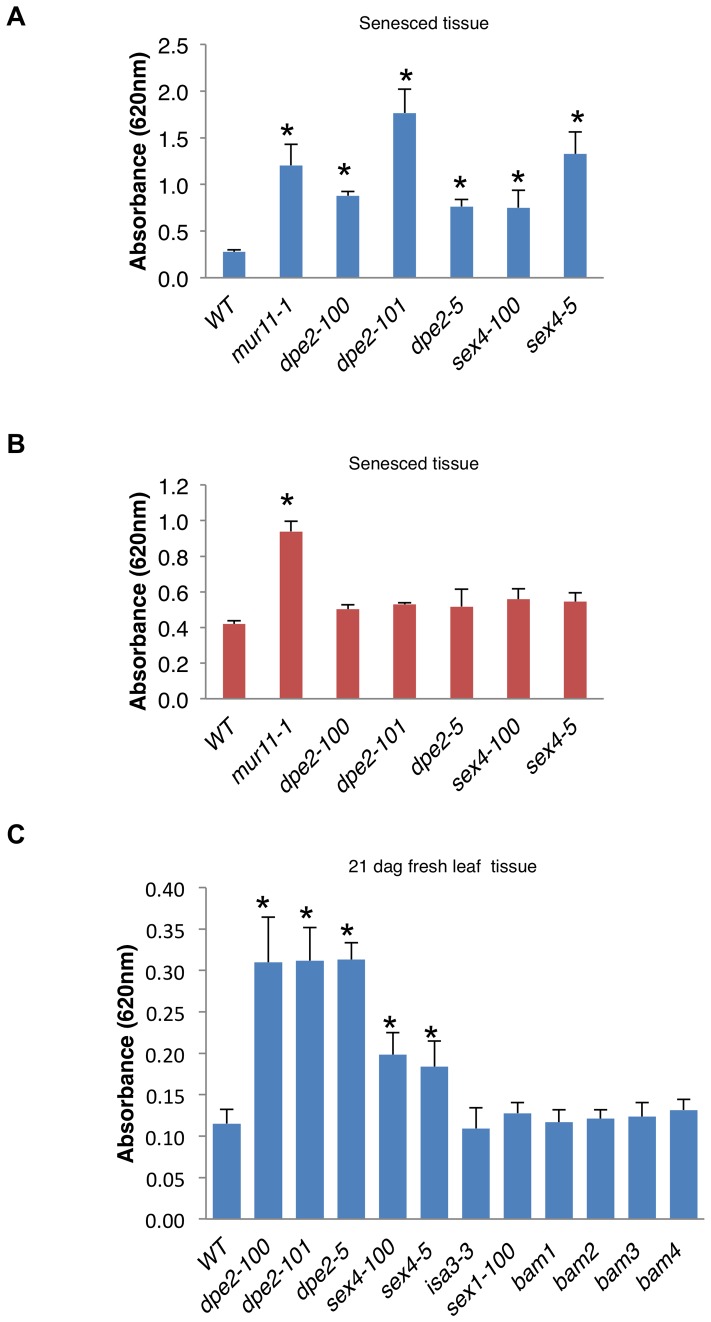
Starch analysis of *rah* mutants *mur11*, *dpe2* and *sex4*. (A) Senesced stem tissue from starch mutants was treated with α-amylase and the amount of sugar from starch released was quantified using the anthrone method. Graph shows absorbance at 620 nm per 0.25 mg dry tissue; values are averages ± s.d. (*n* = 4). We repeated all experiments two times with similar results. (B) Post-amylase treatment, tissue from (A) was assayed by acid hydrolysis for residual sugar release using 1 M H_2_SO_4_. Graph shows absorbance at 620 nm per 0.25 mg dry tissue; values are averages ± s.d. (*n* = 3). (C) Acid hydrolysis of fresh leaf disc tissue from known starch mutants using 1 M H_2_SO_4_. Graph shows absorbance at 620 nm for ¼ of a leaf disc hydrolysate; values are averages ± s.d. (*n* = 4). We repeated all experiments at least three times with similar results. *, P<0.01 using Student's *t*-test.

To understand the molecular nature of the mutant category that showed both a high saccharification and increased starch accumulation we map-based cloned the mutant alleles from three lines (*rah1*, *rah22* and *rah9*). The *rah1* and *rah22* lines contained allelic mutations in the *DISPROPORTIONATING ENZYME 2* (*DPE2*) gene, which encodes a glucosyltransferase required for starch degradation and these lines were subsequently redesignated *dpe2-100* and *dpe2-101* respectively (Table S2). Subsequent molecular analysis of lines *rah3*, *rah5*, *rah14*, *rah21* showed they were siblings of *rah1*. The *rah9* line was a new allele of *STARCH EXCESS 4* (*sex4-100*), which encodes a glycan phosphatase involved in starch degradation (Table S2) [17], [18]. We validated the identification of these genes by showing that T-DNA knockout insertion alleles in both *DPE2* and *SEX4* also showed improved sugar release by acid hydrolysis (Fig. 3A).

The identification of *dpe2* and *sex4* in our screens suggested to us that starch could be a source of acid-dependent sugar release. We determined the contribution of starch to saccharification by treating senesced stem tissue with α-amylase, which specifically converts starch to glucose and maltose (Fig. 3A). Once tissue was devoid of starch, it was subjected to acid hydrolysis to determine the residual sugar release (Fig. 3B). This analysis clearly showed that most of the improved sugar release observed in both *dpe2* and *sex4* mutants can be accounted for by their increased starch content. By contrast, the *mur11-1* samples showed a higher sugar release than wild type even after α-amylase treatment suggesting some of the increased saccharification is due to polymers other than starch.

Finally to further explore the connection of starch overaccumulation and increased saccharification by acid hydrolysis, we subjected a collection of well characterized *Arabidopsis* starch mutants to the acid hydrolysis assay. Our analysis included *starch-excess 1* (*sex1*), which is defective in the regulation of starch degradation [19], *isoamylase 3* (*isa3*), which is defective in a starch debranching enzyme [20], and *β-amylase* (*bam*) mutants, which are defective in the breakdown of starch (*bam1* through *4*) (Fig. 3C) [21]. Surprisingly, only alleles of *mur11*, *dpe2* and *sex4* mutants showed increased sugar release. Together, these results indicate that generically altering starch metabolism does not always result in altered acid dependent sugar release.

### Inhibiting polar auxin transport improves saccharification

**Figure 4:**
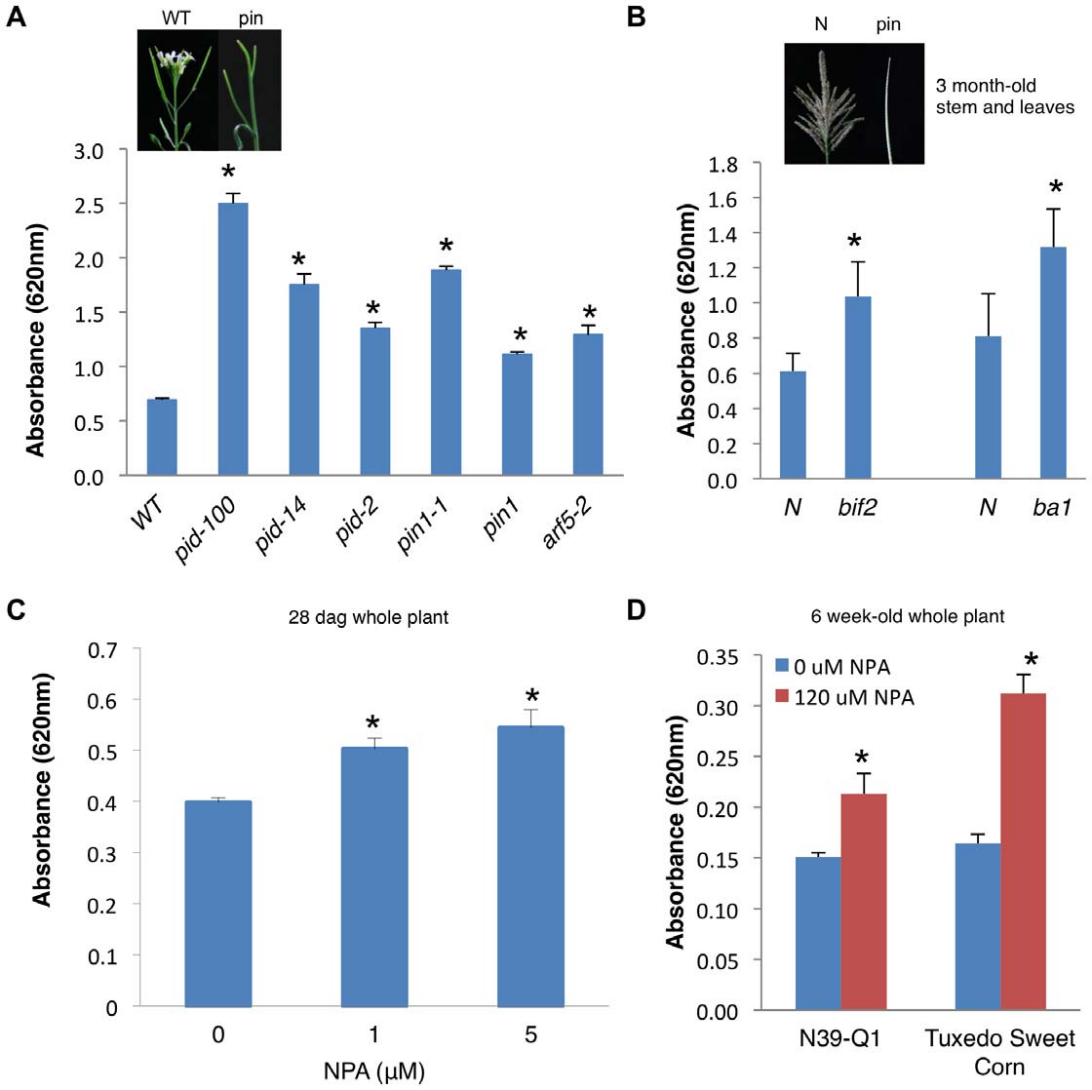
*pin* mutations and NPA treatment results in increased saccharification in *Arabidopsis* and maize. (A) Sugar release measured using anthrone method from ground, senesced stem tissue from *Arabidopsis* pin-shaped inflorescence mutants subjected to 0.2 M acid hydrolysis. Graph shows absorbance at 620 nm per 0.25 mg dry tissue; values are averages ± s.d. (*n* = 3). We repeated all experiments three times with similar results. Inset shows representative pin-shaped inflorescence in *Arabidopsis*. (B) Sugar release measured using anthrone method from ground, stems and leaves of 3 month-old maize inflorescence mutants, *bif2* and *ba1*, subjected to 0.2 M H_2_SO_4_ acid hydrolysis. Graph shows absorbance at 620 nm per 0.25 mg dry tissue; values are averages ± s.d. (*n* = 3–4). N, phenotypically normal siblings. Inset shows representative maize inflorescence mutant. (C) Wild type *Arabidopsis* 28 day-old seedlings grown on MS media supplemented with 0, 1 or 5 µM NPA and subjected to 0.2 M H_2_SO_4_ acid hydrolysis. Graph shows absorbance at 620 nm per 0.25 mg dry tissue; values are averages ± s.d. (*n* = 4). We repeated all experiments two times with similar results. (D) Two maize cultivars were grown for 4 weeks and then treated with 120 µM NPA for 2 weeks and the stems and leaves of the plant was subjected to 0.2 M H_2_SO_4_ acid hydrolysis and sugars released measured using the anthrone method. Graph shows absorbance at 620 nm per 0.25 mg dry tissue; values are averages ± s.d. (*n* = 3–6). *, P<0.01 using Student's *t*-test.

Among those lines which showed improved sugar release in both fresh and senesced, stem tissue, one line in particular (*rah20*) stood out because during reproductive growth it showed an incompletely penetrant pin-shaped inflorescence phenotype. Thus, although we attempted to bias our screen away from mutations that influence development *rah20* did show a post flowering phenotype that was reminiscent of mutations that perturb the polar transport of the plant hormone auxin [22]. Subsequent molecular analysis of this line identified a mutation in the *PINOID* (*PID*) gene (Fig. S3C, Table S2). *PID* encodes a serine-threonine protein kinase that is thought to play a role in the cellular localization of the PIN efflux auxin carrier [23]. We also found that mutations in other genes that result in a pin-shaped phenotype such as *pin1* [22] and *mp* (also known as *arf5*) [24] show an improved saccharification phenotype (Fig. 4A). By contrast, other *auxin response factor* (*arf*) mutants defective in auxin signalling (*arf 6,7,8* and *19*), did not show increase sugar release; however, these mutants also do not have the pin-inflorescence phenotype. Furthermore, none of the single, double or triple combinations of *arf* mutants tested displayed an increase in acid dependent sugar release (Fig. S5). Finally, we tested maize mutants with barren inflorescence phenotypes. *BARREN INFLORESCENCE2* (*BIF2*) is a co-ortholog of *PID* in *Arabidopsis* [13] and *BARREN STALK1* (*BA1*), a basic helix-loop-helix transcription factor, has been shown to be a downstream target of BIF2 in maize [25]. Consistent with the results from *Arabidopsis*, both *bif2* and *ba1* maize inflorescence mutants show an improved saccharification phenotype (Fig. 4B).

The connection between auxin transport and increased sugar release was further probed using a specific inhibitor of auxin transport *N*-1-naphthylphthalamic acid (NPA) [26], [27]. Application of varying concentrations of NPA to wild type *Arabidopsis* seedlings resulted in a 1.5 to 2 fold increase in the release of sugars relative to untreated plants (Fig. 4C). More importantly, the ability to chemically perturb auxin transport allowed us to expand our analysis again to *Zea mays* (maize). Application of NPA to two different cultivars of maize also resulted in a significant increase in saccharification (Fig. 4D). Together, these results provide strong support that genetic or chemical manipulation of auxin transport can increase sugar release from plant biomass.

## Discussion

Most approaches to genetically improving conversion of plant biomass into a fermentable sugar source take advantage of our understanding of cell wall polymer synthesis. This usually involves manipulating glycosyltransferases and glycan synthases that are involved in polymerizing polysaccharides or modulating levels of lignin [28]–[30]. However, our rudimentary knowledge about the regulation of this complex matrix limits this approach. For example, estimates of over 1000 cell wall proteins in *Arabidopsis* alone make it difficult to know which ones will functionally influence saccharification [31]. Furthermore, over 700 genes are annotated as encoding putative glycosyltransferases or glycosyl hydrolases. The goal of this study was to develop a simple, high throughput forward genetic screen to identify genotypes that showed improved sugar release under mild acid treatment and to this end we identified a small collection of of potentially informative lines. The frequency of mutant identification (0.3%) and lack of many alleles within our collection suggests the screen was not saturated and that more genetic variation remains to be discovered. Given the scope of the work here, we limited our analysis to a few assays and we focused on only those mutants belonging to two categories. We have therefore not fully characterized a large number of mutants in our collection and these may yet reveal more interesting information about accessible sugars originating from either starch or the cell wall. The phenotypes of mutants with sensitivity to acid and/or enzymatic hydrolysis or ectopic ligninification, might suggest that apart from starch metabolism a number of different aspects of cell wall integrity might have also been perturbed. Although there are many possibilities, our approach might have, for example, enriched for mutations in genes that affect pectin structure. In the context of biomass conversion this would be fortuitous, since there is growing evidence which suggests that pectins are crosslinked with hemicelluloses, phenolics and cell wall proteins (see [32] and references within). Indeed the modification of pectins can lead to improved saccharification of plant biomass; presumably by reducing occlusion of cellulose and hemicellulose by pectins, thus increasing the accessibility of cellulases and hemicellulases to their substrates [33].

The observation that *MUR11*, a gene that was originally found through a focussed cell wall genetic screen, was identified in our screens does demonstrate the utility of using weak acid hydrolysis to genetically probe for the saccharification properties of plant biomass. In addition, a clear advantage of using forward genetic screens, which can identify point mutations or chromosomal rearrangements, is that the types of mutations that are identified are not limited to loss of function alleles; reduction of function or gain of function mutations can also be identified. From the perspective of biomass production it is often better to identify weak alleles that have no deleterious effects on plant growth but that do lead to improved extraction of sugar. For example, the weak *mur11-1* allele is more useful than the knock-out allele, *sac9-3*, which leads to dwarfed plants, since the *mur11-1* allele leads to equivalent improvement of sugar extraction without severely affecting plant growth and in turn biomass. Whether modification of genes such as *MUR11* will find utility in the farmer's field is still a matter of contention. Nevertheless, it is only by obtaining an allelic series that it will be possible to determine how much of a factor developmental defects have on the saccharification properties of specific source material.

The identification of mutants that overaccumulate starch in vegetative tissues presents an unforeseen approach with respect to the improvement of fermentable sugars for biofuel production; an observation that has been made by others [34]. Because starch is a simple easily accessible glycopolymer compared to lignocellulose, it is efficiently converted to sugar for ethanol production. However, unlike reproductive tissues such as corn kernels, starch levels in stems and leaves are limited and therefore these vegetative tissues are not considered a useful starch based feedstock. Here we show that genetically increasing vegetative starch levels can contribute to the overall fermentable sugar yields during acid pretreatment or even by enzyme hydrolysis. Because this sugar source is not lignocellulosic, in principle its genetic manipulation should be a stackable trait with lignocellulosic feedstock technologies. The observation that only some starch excess mutants were identified in our screens, however, suggests the relationship between starch and acid-dependent sugar release is complex. Possibly, certain mutants accumulate starch as a secondary consequence of a mutation. For example, not all sugar release from *mur11* mutants is explained through starch accumulation, which is consistent with this mutant also having other defects. It is also possible that various starch accumulating mutants accumulate slightly different forms of starch and that these forms may not be equally accessible to acid hydrolysis.

An association between cell walls and auxin has existed for some time with respect to the role of this hormone in cell expansion [35]. More recently, the demonstration that mutating the cellulose synthase gene *CESA3* results in mislocalization of PIN1 efflux carriers further suggests a close linkage between auxin transport and cell wall synthesis [36]. The observation that *pinoid* and additional pin-shaped inflorescence mutants have increased sugar release again suggests a role for auxin in influencing sugar accessibility. Interestingly, this association is limited to auxin mutants that display a pin-shaped inflorescence phenotype, which may mean that altering cell wall integrity contributes to aberrant inflorescence development. Alternatively, auxin transport may influence the accumulation of cytosolic sugar or starch in vegetative tissues and lead to better saccharification. At this point, what the underlying mechanism(s) are that are contributing to the increased saccharification are a matter of speculation.

Our acid hydrolysis screen only identified *pinoid* loss-of-function mutants. Presumably, additional *Arabidopsis* mutants that form pin-shaped inflorescences such as *pin1* or *mp* were not found because unlike *pinoid* these mutants are completely penetrant and therefore infertile. Although this makes propagation of these lines problematic, the pin-shaped phenotype may have advantages with respect to preventing gene flow among commercially grown transgenic crops. Related to this, our studies show that treatment of wild type *Arabidopsis* and maize plants with the polar auxin transport inhibitor, NPA, also results in increased saccharification. In contrast to making transgenic plants, which can be costly, time-consuming and often involve constitutive phenotypes, chemically-induced phenotypes using compounds such as NPA should allow more tailored temporal and spatial control of biomass composition. Moreover, NPA, which is already an approved pre-emergence herbicide, can be applied broadly to crops that have rudimentary genetics or that are difficult to transform. Finally, the ability to increase saccharification using NPA suggests chemical genetic screening using *Arabidopsis* can be applied to develop chemical leads that might be useful in biomass pretreatment processing [12].

## Supporting Information

Figure S1 (A) Boxplot of 100 wild type *Arabidopsis* leaf discs subjected to 1 M H2SO4 treatment at room temperature for one hour. The bold horizontal line represents the median which has an absorbance reading of 0.1156 at 620 nm. Error bars show ± standard deviation. (B) Absorbance readings from anthrone acid hydrolysis are quantified against a glucose curve. Candidate *rah* mutants are considered as releasing a significant amount of sugars when readings measure 2 or more standard deviations above wild type (mean Abs_6200 nm_ 0.116±0.002).
(TIF)

Figure S2 Absorption spectra of pure sugars at different concentrations after 5 min heating with anthrone reagent.
(TIF)

Figure S3 Map based cloning of *RAH* genes. CAPS and/or SSLP markers (indicated by their position in Mb) were used to narrow down the interval and corresponding BAC. Number of recombinants at each position indicated. For fine mapping, we used 172 plants for *DPE2*, 201 plants for *PID* and 144 plants *SEX4*. (A) Two alleles, *rah1* and *rah22*, of *DISPROPORTIONATING ENZYME 2* (*DPE2*) were cloned, a glucosyltransferase required for starch degradation by metabolizing maltose ^12^. The *dpe2-100* allele contains a G-to-A base pair change resulting in a premature stop codon in exon 6 and the *dpe2-101* allele contains a G-to-A base pair change resulting in the conversion of an arginine residue to a lysine residue in exon 13 (Table S2). (B) The *rah20* mutant, which had a pin-shaped inflorescence phenotype encodes *PINOID* (*PID*), a serine-threonine protein kinase ^18^ that plays a role in PIN-FORMED protein localization ^34^. A G-to-A base pair change in *pid-100* results in the conversion of an aspartate residue to an asparagine residue (Table S2), an invariant protein kinase residue ^18^. (C) We cloned *STARCH-EXCESS4* (*SEX4*), a protein phosphatase required for starch degradation ^35^. The *sex4-100* allele has a G to A base pair change at the 5′ splice junction of intron 8 (Table S2).
(TIF)

Figure S4 Acid hydrolysis of mutants of the SAC domain family of *Arabidopsis*. The following T-DNA insertions were used: *sac1-1* (SALK_070875), *sac1-2* (SALK_020109), *sac2-1* (SALK_099031), *sac2-2* (SALK_091926), *sac3-1* (SALK_023548), *sac3-2* (SALK_049623), *sac4-1* (SALK_119184), *sac4-2* (SALK_005871), *sac4-3* (SALK_056500), *sac5-1* (SALK_012372), *sac5-2* (SALK_125856), *sac6-1* (SALK_021488), *sac6-2* (SALK_136049), *sac7-1* (SALK_000558), *sac7-2* (SALK_092575), *sac8-1* (SALK_062145) and *sac8-2* (SALK_115643). Leaf disc tissue from 21 day-old plants was assayed using 1 M H_2_SO_4_. Graphs show absorbance at 620 nm for ¼ of leaf disc hydrolysate; values are averages ± s.d. (*n* = 3–4). *, P<0.01 using Student's *t*-test.
(TIF)

Figure S5 Acid hydrolysis of *auxin response factor* mutants of *Arabidopsis*. Leaf disc tissue from 21 day-old plants was assayed using 1 M H_2_SO_4_. Graphs show absorbance at 620 nm for ¼ of leaf disc hydrolysate; values are averages ± s.d. (*n* = 4–8).
(TIF)

Table S1 Known cell wall mutants and their gene products. *MUR11* was molecularly identified in this study and is highlighted in red.
(XLS)

Table S2 Genotypes used in this study.
(XLS)

Table S3 Monosaccharide composition of *rah* mutants by gas chromatography.
(XLS)

Table S4 Acid hydrolysis and enzymatic assays of *rah* mutants.
(XLS)
