# Supplementary figures and images for: Forward Genetic Screening for the Improved Production of Fermentable Sugars from Plant Biomass
Source: PLoS One. 2013 Jan 31;8(1):e55616. doi: 10.1371/journal.pone.0055616 (PMC3561329; doi:10.1371/journal.pone.0055616)

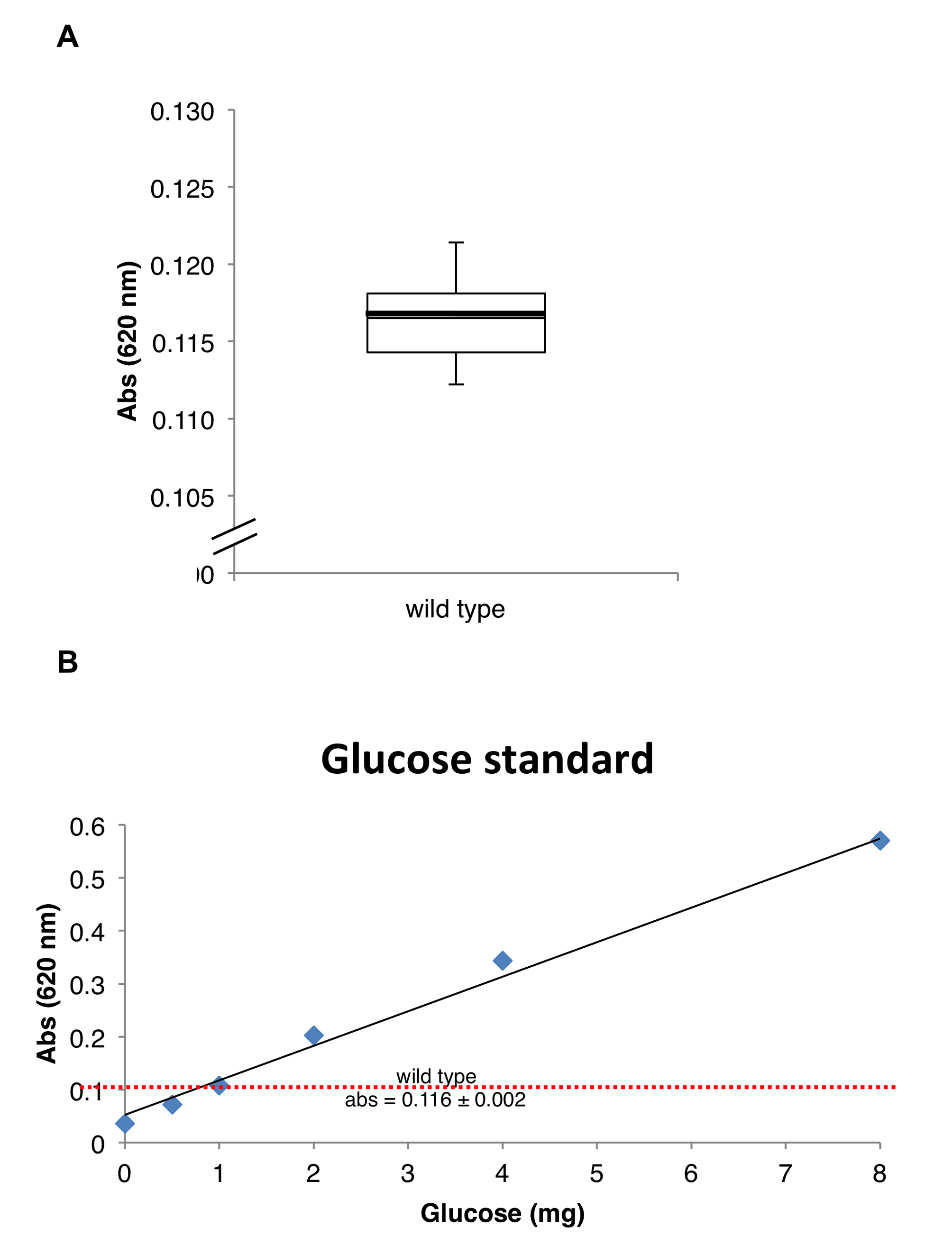

Supplement: Figure S1 — (A) Boxplot of 100 wild type Arabidopsis leaf discs subjected to 1 M H2SO4 treatment at room temperature for one hour. The bold horizontal line represents the median which has an absorbance reading of 0.1156 at 620 nm. Error bars show ± standard deviation. (B) Absorbance readings from anthrone acid hydrolysis are quantified against a glucose curve. Candidate rah mutants are considered as releasing a significant amount of sugars when readings measure 2 or more standard deviations above wild type (mean Abs6200 nm 0.116±0.002). (TIF) [file pone.0055616.s001.tif]

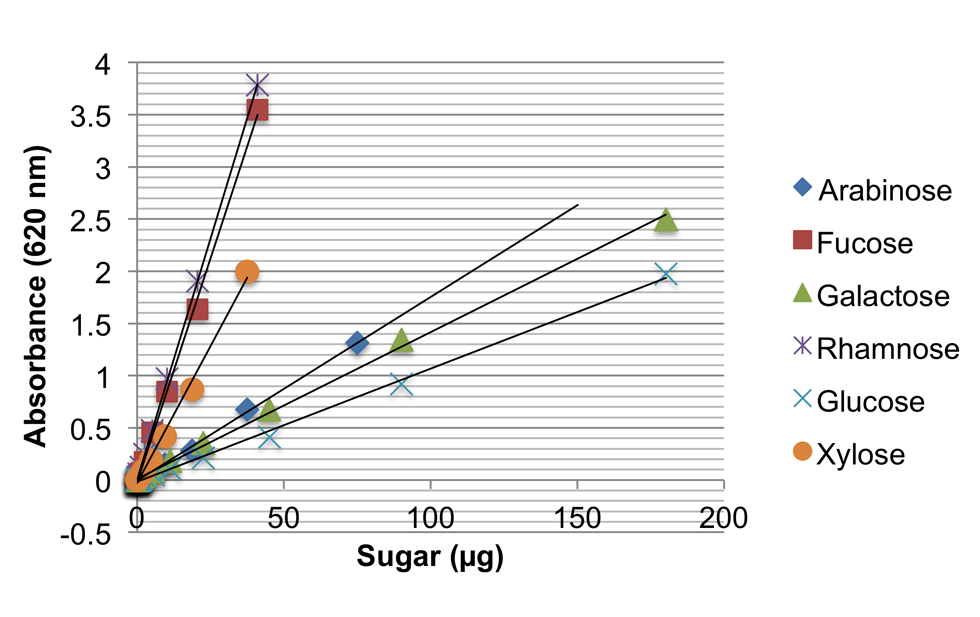

Supplement: Figure S2 — Absorption spectra of pure sugars at different concentrations after 5 min heating with anthrone reagent. (TIF) [file pone.0055616.s002.tif]

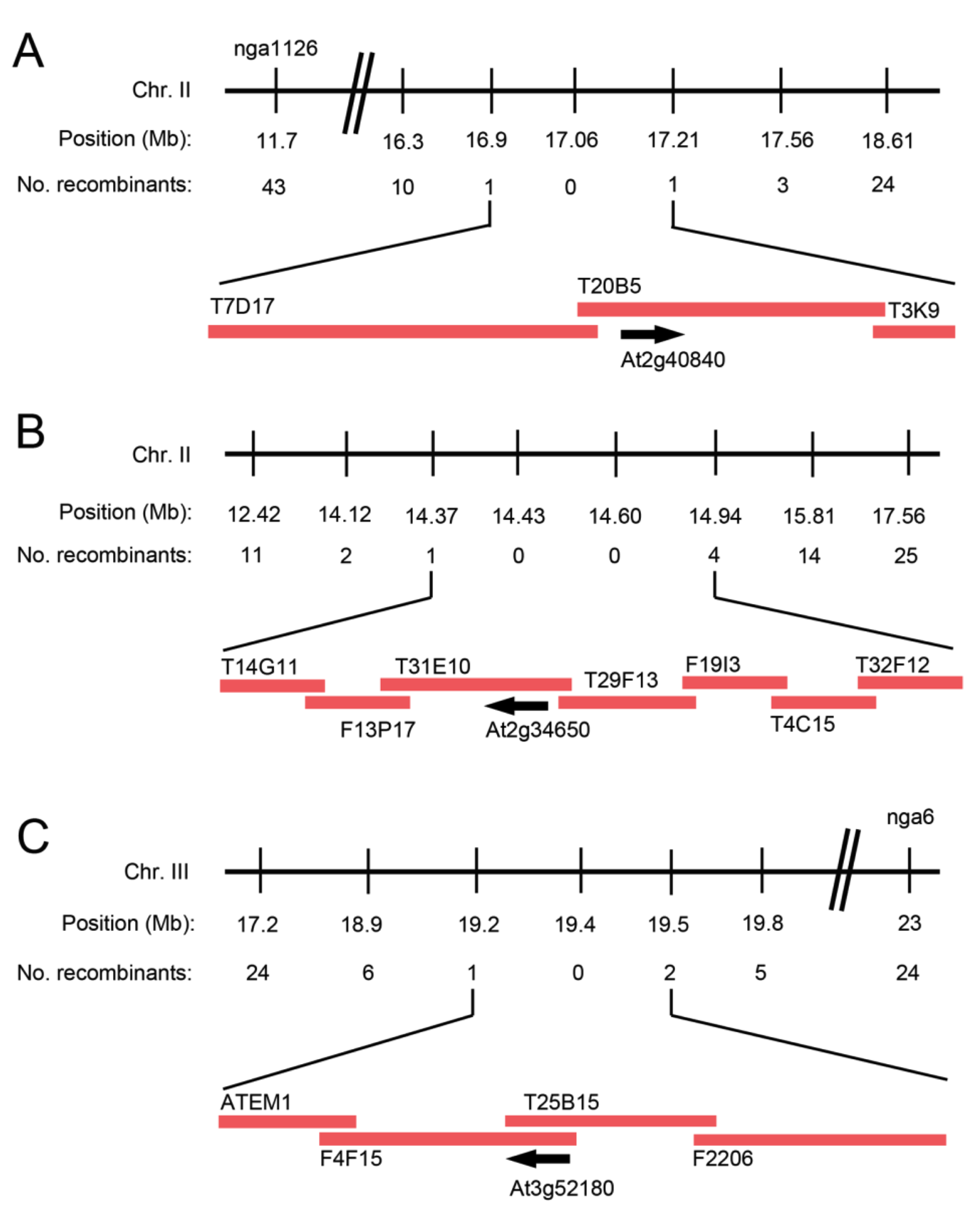

Supplement: Figure S3 — Map based cloning of RAH genes. CAPS and/or SSLP markers (indicated by their position in Mb) were used to narrow down the interval and corresponding BAC. Number of recombinants at each position indicated. For fine mapping, we used 172 plants for DPE2, 201 plants for PID and 144 plants SEX4. (A) Two alleles, rah1 and rah22, of DISPROPORTIONATING ENZYME 2 (DPE2) were cloned, a glucosyltransferase required for starch degradation by metabolizing maltose 12. The dpe2-100 allele contains a G-to-A base pair change resulting in a premature stop codon in exon 6 and the dpe2-101 allele contains a G-to-A base pair change resulting in the conversion of an arginine residue to a lysine residue in exon 13 (Table S2). (B) The rah20 mutant, which had a pin-shaped inflorescence phenotype encodes PINOID (PID), a serine-threonine protein kinase 18 that plays a role in PIN-FORMED protein localization 34. A G-to-A base pair change in pid-100 results in the conversion of an aspartate residue to an asparagine residue (Table S2), an invariant protein kinase residue 18. (C) We cloned STARCH-EXCESS4 (SEX4), a protein phosphatase required for starch degradation 35. The sex4-100 allele has a G to A base pair change at the 5′ splice junction of intron 8 (Table S2). (TIF) [file pone.0055616.s003.tif]

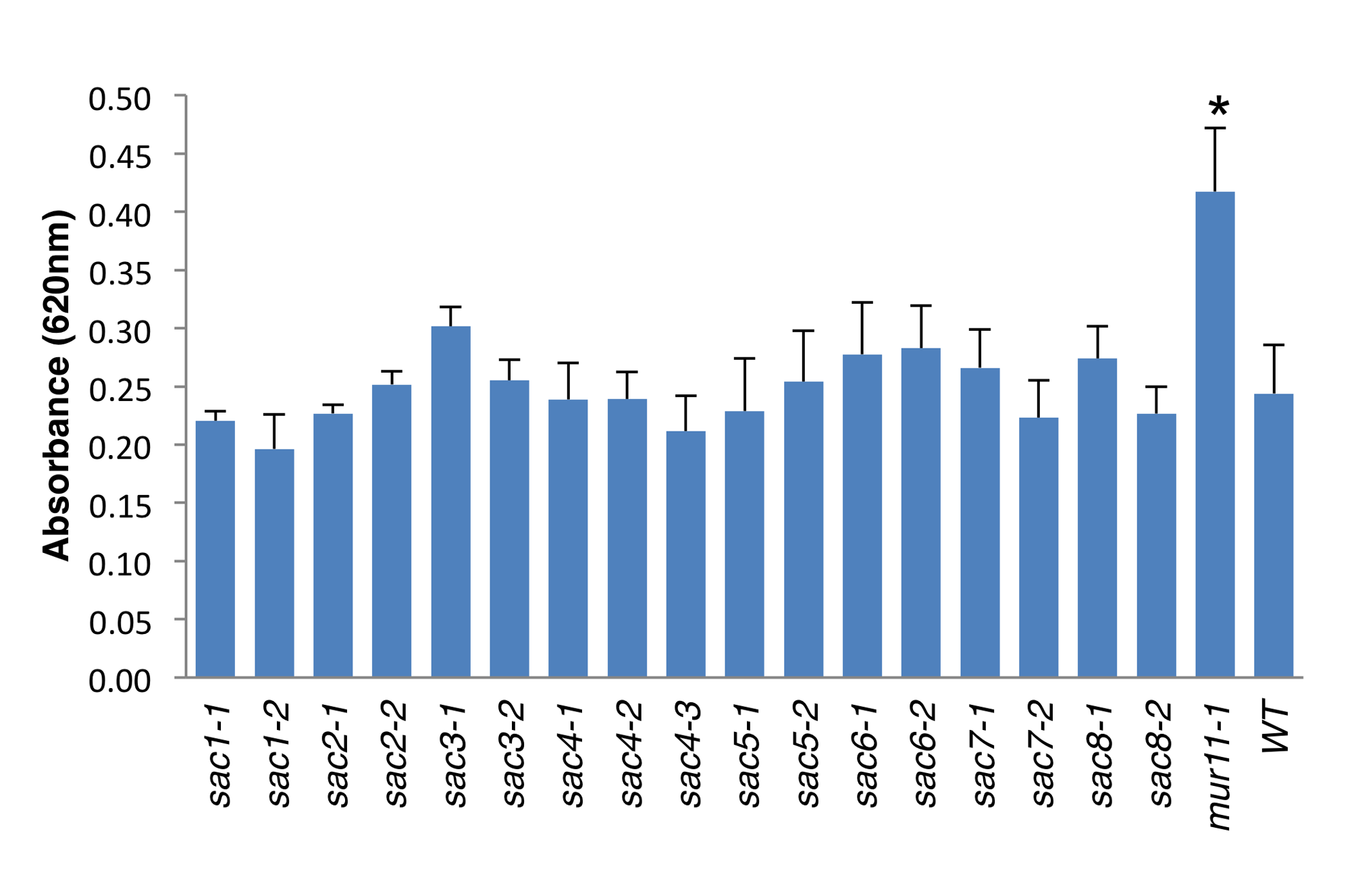

Supplement: Figure S4 — Acid hydrolysis of mutants of the SAC domain family of Arabidopsis. The following T-DNA insertions were used: sac1-1 (SALK_070875), sac1-2 (SALK_020109), sac2-1 (SALK_099031), sac2-2 (SALK_091926), sac3-1 (SALK_023548), sac3-2 (SALK_049623), sac4-1 (SALK_119184), sac4-2 (SALK_005871), sac4-3 (SALK_056500), sac5-1 (SALK_012372), sac5-2 (SALK_125856), sac6-1 (SALK_021488), sac6-2 (SALK_136049), sac7-1 (SALK_000558), sac7-2 (SALK_092575), sac8-1 (SALK_062145) and sac8-2 (SALK_115643). Leaf disc tissue from 21 day-old plants was assayed using 1 M H2SO4. Graphs show absorbance at 620 nm for ¼ of leaf disc hydrolysate; values are averages ± s.d. (n = 3–4). *, P<0.01 using Student's t-test. (TIF) [file pone.0055616.s004.tif]

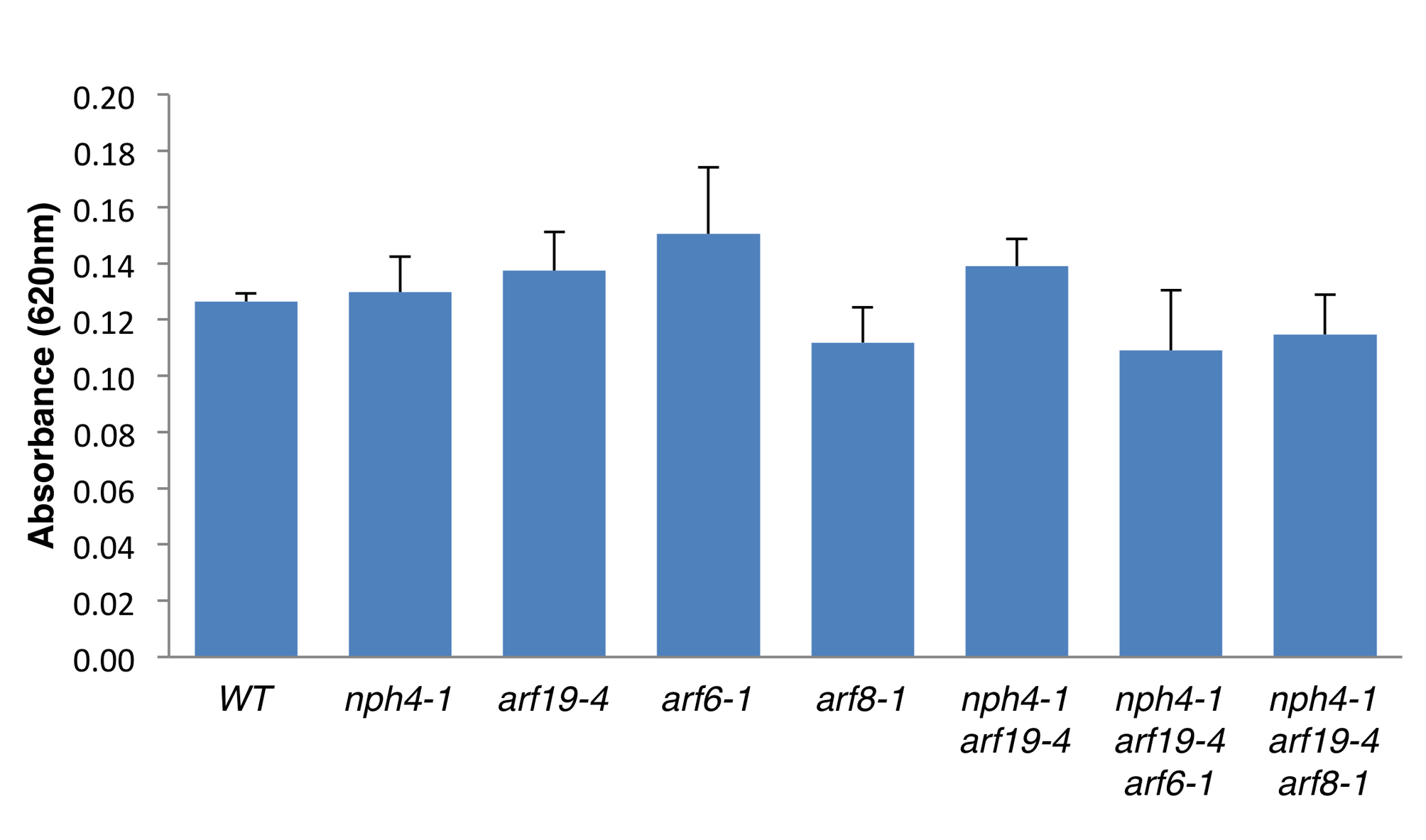

Supplement: Figure S5 — Acid hydrolysis of auxin response factor mutants of Arabidopsis. Leaf disc tissue from 21 day-old plants was assayed using 1 M H2SO4. Graphs show absorbance at 620 nm for ¼ of leaf disc hydrolysate; values are averages ± s.d. (n = 4–8). (TIF) [file pone.0055616.s005.tif]
